# Rosiglitazone Does Not Affect the Risk of Inflammatory Bowel Disease: A Retrospective Cohort Study in Taiwanese Type 2 Diabetes Patients

**DOI:** 10.3390/ph16050679

**Published:** 2023-05-01

**Authors:** Chin-Hsiao Tseng

**Affiliations:** 1 Department of Internal Medicine, National Taiwan University College of Medicine, Taipei 10051, Taiwan; ccktsh@ms6.hinet.net; 2 Division of Endocrinology and Metabolism, Department of Internal Medicine, National Taiwan University Hospital, Taipei 10002, Taiwan; 3 National Institute of Environmental Health Sciences of the National Health Research Institutes, Zhunan 35053, Taiwan

**Keywords:** Crohn’s disease, inflammatory bowel disease, pharmacoepidemiology, rosiglitazone, Taiwan, type 2 diabetes mellitus, ulcerative colitis

## Abstract

Human studies on the effect of rosiglitazone on inflammatory bowel disease (IBD) are still lacking. We investigated whether rosiglitazone might affect IBD risk by using the reimbursement database of Taiwan’s National Health Insurance to enroll a propensity-score-matched cohort of ever users and never users of rosiglitazone. The patients should have been newly diagnosed with diabetes mellitus between 1999 and 2006 and should have been alive on 1 January 2007. We then started to follow the patients from 1 January 2007 until 31 December 2011 for a new diagnosis of IBD. Propensity-score-weighted hazard ratios were estimated with regards to rosiglitazone exposure in terms of ever users versus never users and in terms of cumulative duration and cumulative dose of rosiglitazone therapy for dose–response analyses. The joint effects and interactions between rosiglitazone and risk factors of psoriasis/arthropathies, dorsopathies, and chronic obstructive pulmonary disease/tobacco abuse and the use of metformin were estimated by Cox regression after adjustment for all covariates. A total of 6226 ever users and 6226 never users were identified and the respective numbers of incident IBD were 95 and 111. When we compared the risk of IBD in ever users to that of the never users, the estimated hazard ratio (0.870, 95% confidence interval: 0.661–1.144) was not statistically significant. When cumulative duration and cumulative dose of rosiglitazone therapy were categorized by tertiles and hazard ratios were estimated by comparing the tertiles of rosiglitazone exposure to the never users, none of the hazard ratios reached statistical significance. In secondary analyses, rosiglitazone has a null association with Crohn’s disease, but a potential benefit on ulcerative colitis (UC) could not be excluded. However, because of the low incidence of UC, we were not able to perform detailed dose–response analyses for UC. In the joint effect analyses, only the subgroup of psoriasis/arthropathies (-)/rosiglitazone (-) showed a significantly lower risk in comparison to the subgroup of psoriasis/arthropathies (+)/rosiglitazone (-). No interactions between rosiglitazone and the major risk factors or metformin use were observed. We concluded that rosiglitazone has a null effect on the risk of IBD, but the potential benefit on UC awaits further investigation.

## 1. Introduction

Inflammatory bowel disease (IBD) is a chronic relapsing inflammatory disease of the intestinal tract mediated by immunity. It may have varying courses and complications, and both the innate immune system and the adaptive immune system can be involved [1,2,3]. Proinflammatory immune mediators such as interleukin 17, interleukin 23, interferon gamma, and tumor necrosis factor alpha are always excessively expressed [4,5,6].

IBD is generally classified as Crohn’s disease (CD) and ulcerative colitis (UC) [7] and the clinical manifestations may include watery diarrhea, fatigue, weight loss, abdominal pain, and bleeding [4]. Diarrhea may be insidious and episodic and can occur intermittently for many years before IBD is diagnosed, and patients may have suffered from significant body weight loss and malnutrition at the time of its diagnosis [4]. Intestinal fistulas to adjacent structures such as bowel, vagina, bladder, or skin can happen in 20–40% of the patients with CD [4]. Perianal fistula is associated with a more aggressive phenotype of the disease [4]. Chronic inflammation may lead to the development of strictures and intestinal obstruction [4]. Fever is usually low grade but higher fever may indicate more severe inflammation or is a sign of abscess formation or perforation [4]. Sometimes IBD can be life-threatening because of severe bleeding in 1–2% of the patients [4,8].

Extraintestinal involvement of skin, joints, eyes, liver, bile ducts, kidney, bone, and cardiovascular system can be seen in up to half of the patients [4,9,10,11]. Furthermore, IBD may increase the risk of colorectal cancer [12,13] and patients with IBD may have a higher incidence of atherosclerotic cardiovascular diseases, heart failure, atrial fibrillation [14], psoriasis [15], Alzheimer’s disease [16,17], depression, and anxiety [18,19,20].

Because biomarkers, such as C-reactive protein, fecal calprotectin, interleukins, tumor necrosis factor alpha, antibodies, etc., are nonspecific and clinical presentations always have difficulty differentiating between the two disease entities of CD and UC, laboratory examinations such as colonoscopy, ultrasonography, computed tomography enterography, or magnetic resonance enterography are necessary for aiding in the diagnosis of IBD [4,21,22].

Some type of colitis may occur in approximately 10–15% of the population [8]. The highest prevalence rates of IBD (approximately 0.3%) are reported in Europe and North America, but its incidence seems to be stable or decreasing in these countries [23]. The incidence and prevalence of IBD in Asia, South America, and Africa are usually lower than those observed in Western countries [2,23,24,25,26]. However, the incidence of IBD has been increasing in these newly industrialized countries since 1990s [2,23,24,25,26]. In South Korea, IBD prevalence and incidence in 2015 were approximately 108.4 per 100,000 population and 9 per 100,000 population, respectively [27]. IBD increased by approximately 2.3% from 2010 to 2019 in South Korea [28]. In Taiwan, the respective prevalence and incidence rates were 16.7 and 1.4 per 100,000 population in 2015 [27], and the annual percentage change in the increasing incidence of IBD from 1998 to 2008 has been estimated to be 4% to 5% [23]. In China, it was reported that the age-standardized incidence and prevalence both increased by approximately 2.5-fold within a period of 30 years from 1990 to 2019 [29]. Asian immigrants to Western countries also experience an increasing incidence of IBD [30].

Though not fully elucidated, the etiology of IBD involves the interplay among host, microbiota, and environmental factors [1,2,8,31,32,33,34,35,36]. Researchers have identified more than 230 genetic loci associated with IBD. These genes are primarily involved in major histocompatibility complex, pattern recognition, inflammation, and apoptosis [4,37,38,39]. Environmental risk factors relating to industrialization and excessive sanitation and hygiene have been reported. More specifically, risk factors may include metabolic syndrome, lack of exercise, work shift, psychological stress, vitamin D deficiency, and dietary patterns (more consumption of calorically dense diet, animal protein, high-fat diet and high-sugar diet and less intake of fiber-containing vegetables, fruits, cereals, and nuts) [2,37,40,41,42,43]. In addition, milk formula feeding, history of childhood infection and vaccination, and medications such as antibiotics, nonsteroidal anti-inflammatory drugs, and oral contraceptives have also been reported [2,37,40,41,42,43]. On the other hand, breastfeeding is protective against IBD [2]. Studies also suggested that cigarette smoking and appendectomy both aggravate CD but may alleviate UC [2]. Gut microbiota are pivotal in the development of IBD because they may produce metabolites that affect the hosts’ immune response and control the release of inflammatory cytokines [44].

The peroxisome proliferator–activator receptors (PPARs) belong to the nuclear hormone receptors’ superfamily which contains three isoforms, i.e., PPARα, PPARβ/δ, and PPARγ [1,8]. They act as transcription factors that activate the expression of various genes [1]. PPARγ is abundantly expressed in colonic epithelial cells and exerts antiproliferative, anti-inflammatory, and immune modulating effects [1,8,45,46]. The usefulness of PPARγ in the treatment of IBD has long been investigated in preclinical studies [1,8,47,48,49,50,51,52]. Emodin is a Chinese herb drug that has been used to treat IBD. Its potential mode of action is through the activation of PPARγ-related signaling [53].

A class of oral antidiabetic drugs known as thiazolidinedione (TZD) improves insulin resistance by targeting PPARγ. In Taiwan, only two drugs in the class, i.e., rosiglitazone and pioglitazone, have been marketed [54]. However, rosiglitazone has been withdrawn from the market in many countries, including Taiwan, following the publication of a meta-analysis in 2007 that suggested a significantly higher risk of cardiovascular disease [55]. Pioglitazone survived the market even though a signal of increased risk of bladder cancer was raised in 2011 [56].

To our knowledge, there are scanty population-based studies investigating the potential role of antidiabetic drugs in the class of TZD in the prevention of IBD in humans. In our recent study, we found a null association between pioglitazone (the only TZD currently available in Taiwan) exposure and IBD risk in Taiwanese patients with type 2 diabetes mellitus [57]. Although rosiglitazone is not currently used in clinical practice in Taiwan, it remains a clinically important issue to look into the potential usefulness of rosiglitazone in the prevention of an intractable disease of IBD. In the present study, we investigated IBD risk with regard to rosiglitazone exposure in a cohort of patients with type 2 diabetes mellitus matched on propensity score by using the reimbursement database derived from the nationwide National Health Insurance (NHI) in Taiwan.

## 2. Results

As shown in Table 1, ever users and never users of rosiglitazone derived from the NHI database and matched on propensity score (PS) are well balanced in all characteristics because none of the variables showed a value of standardized difference between ever users and never users of rosiglitazone > 10%.

Table 2 shows the incident case numbers, incidence rates, and hazard ratios of IBD in never users of rosiglitazone and in different subgroups of ever users in the primary analyses. All results suggested a nonsignificant association between rosiglitazone and IBD. In secondary analyses, when IBD was analyzed separately for CD and UC, we found that most of the IBD cases were diagnosed as CD (97 cases in never users and 92 cases in ever users) and only 18 cases were diagnosed as UC (15 cases in never users and 3 in ever users). The estimated hazard ratios for CD and UC were 0.964 (95% confidence interval: 0.725–1.282, *p* = 0.8016) and 0.203 (95% confidence interval: 0.059–0.700, *p* = 0.0116), respectively. For the dose–response analyses for cumulative duration and cumulative dose, none of the tertiles reached statistical significance for the CD analyses. Because there were only three cases of UC among ever users, we actually did not have sufficient case numbers for the dose–response analyses for UC in terms of cumulative duration and cumulative dose.

Table 3 shows the results that investigated the joint effects of and interactions between rosiglitazone use and major risk factors of IBD/metformin after adjustment for all covariates listed in Table 1. In the analyses of joint effects, except for the subgroup of psoriasis/arthropathies (-)/rosiglitazone (-) that showed a significantly lower risk in comparison to the subgroup of psoriasis/arthropathies (+)/rosiglitazone (-), none of the other hazard ratios was statistically significant. There was a lack of significant interaction between rosiglitazone and the risk factors/metformin use in any of the models.

## 3. Discussion

### 3.1. Main Findings

The findings of this study did not support any effect of rosiglitazone use on the risk of IBD (Table 2). Furthermore, no interaction was observed between rosiglitazone and any of the risk factors and between rosiglitazone and metformin (Table 3).

### 3.2. Explanations for the Discrepant Findings in Preclinical Studies

In in vitro and in vivo studies, PPARγ may have a potential benefit on IBD through the crosstalk with metabolism and inflammation [1,8,47,48,49,50,51,52,53]. However, there is a lack of evidence to support such a benefit in humans. Our previous study on pioglitazone [57] and the present study on rosiglitazone do not support such a benefit of either TZD on the occurrence of IBD in humans. There are some possible explanations for such discrepancies between preclinical studies and the observational studies conducted in humans.

First, it should be mentioned that colitis in animal models of IBD in preclinical studies is induced mainly by chemicals. Commonly used chemicals include oxazolone, dextran sodium sulphate, dinitrobenzene sulfonic acid, trinitrobenzene sulfonic acid, and intracolonic instillation of acetic acid [8]. Because the pathogenesis of the colitis induced by these chemicals might not be the same as that seen in human IBD, findings derived from in vitro and animal studies should not be directly applied to humans. Colitis induced by chemicals might lead to inflammation following the toxic damages to the colon, which is different from what we know about human IBD that is characterized by inflammation mainly induced by the induction of autoantibodies and the destruction by cytokines.

Second, the doses of rosiglitazone used in in vitro and in vivo studies and the concentrations of rosiglitazone in the medium or in the animals’ blood derived from such administered doses might not be corresponding to the clinically used doses and the blood concentrations that might have been derived in patients with type 2 diabetes mellitus. In clinical trials, rosiglitazone is generally used in a daily dose of 2 to 12 mg [58]. In Taiwan, the generally prescribed daily dose of rosiglitazone is either 4 mg or 8 mg. Whether these clinically used doses of rosiglitazone can be translated to the concentrations used in in vitro or in animal studies [59,60,61] remains to be investigated.

Third, the blood concentration of rosiglitazone derived from oral administration while used for the treatment of hyperglycemia in humans does not guarantee a delivery of sufficient rosiglitazone to the colon for the activation of PPARγ locally. Recent drug development by using nanotechnology [59] or topical administration of rosiglitazone [61] may provide more specific delivery of rosiglitazone to the target organ and tissue and is worthy of more in-depth investigation. Novel PPARγ activators are also being investigated for the treatment of colitis in animals [46].

Fourth, the effects of rosiglitazone and pioglitazone may differ among different species, and the activation of PPARγ by rosiglitazone and pioglitazone in different cells may result in different biological functions, some even counteracting each other, resulting in a variety of different clinical effects. These may explain the different clinical effects of rosiglitazone and pioglitazone observed in different cancer and noncancer diseases in humans. For example, rosiglitazone may adversely affect lipid profile [62] and we did observe a lack of association between rosiglitazone and bladder cancer [63] and dementia [64] but a significantly lower risk of thyroid cancer [65]. On the other hand, pioglitazone improves lipid profiles [62], is associated with a significant risk reduction of dementia [66], and shows a null association with thyroid cancer [67]. Though controversial, a potentially higher risk of bladder cancer associated with pioglitazone use [56] should be cautiously attended. Therefore, rosiglitazone and pioglitazone should be viewed as different entities and they should be investigated separately and not together.

Fifth, though not statistically significant, the overall hazard ratio of 0.870 (95% confidence interval: 0.661–1.144) in Table 2 favored a risk reduction of approximately 13% associated with rosiglitazone use. We could not exclude the possibility of a lack of statistical power and a lack of adjustment for some unmeasured confounders such as microbiota and nutritional and dietary factors in the primary analyses. In secondary analyses, when CD and UC were separately analyzed, although the risk for CD associated with rosiglitazone use was not significant, we did observe a significantly lower risk of UC associated with rosiglitazone. Because there were only three cases of UC among ever users, we did not have sufficient incident case numbers for dose–response analyses for UC. We recognize that the currently available database is not competent to answer whether the effects of rosiglitazone might not be the same for CD and UC, and we cannot completely exclude a possible benefit of rosiglitazone on UC. Future analyses based on an additional request for a larger database from the NHI should be considered to answer these questions.

### 3.3. Potential Explanations for the Discrepant Findings between Metformin and TZDs

The discrepant findings between metformin [68], which shows a significantly reduced risk of IBD, and TZDs including pioglitazone [57] and rosiglitazone (the findings of the present study), which show a null association with IBD, implied some clues to the pathogenesis of IBD in patients with type 2 diabetes mellitus and deserved discussion.

PPARγ is abundantly expressed in the intestinal epithelium, where it plays important roles in maintaining a healthy intestinal tract by inhibiting the expression of inflammatory cytokines activated via either the innate or adaptive immune system [1,2,3]. However, environmental factors such as obesity [42] and compositional changes of the gut microbiota by diet or medications [1] are crucial in the development of IBD. The emergence of these environmental risk factors following the industrialization of our societies and Westernization of our lifestyle may contribute to the increasing trends of IBD in recent years in developing countries, including Taiwan [2,23,24,25,26,27,28,29,30], because genetic mutations may not be responsible for the rapid evolving change of the disease.

Downregulation of PPARγ expression may be triggered by these environmental risk factors, resulting in the activation of the immune-mediated inflammatory processes seen in IBD [1]. Although TZDs used in patients with type 2 diabetes mellitus may theoretically alleviate inflammation via the activation of PPARγ [69], such a benefit has not been well demonstrated in humans. TZDs may significantly increase body weight when used for glycemic control [70] and they do not significantly change the composition of gut microbiota either by rosiglitazone [71,72] or by pioglitazone [73]. Therefore, the anti-inflammatory effect of TZDs might have been offset by the body weight gain following their use.

On the other hand, metformin has some pleiotropic effects that TZDs do not possess. First, metformin does not increase body weight and it may even have a mild weight reduction effect [74]. Second, patients with IBD show a reduced relative abundance of *Akkermansia muciniphila,* and administration of this bacterial species has shown some promising results in the treatment of IBD [75,76,77]. A recent study suggests that metformin significantly increases the relative abundance of *Akkermansia* but pioglitazone fails to do so [78]. Another study shows that rosiglitazone treatment cannot restore the microbiota composition in mice fed with a high-fat diet [72]. *Akkermansia muciniphila* may produce metabolites including propionate and butyrate [76,79,80,81], which are important in amplifying the PPARγ transcriptional activities involving in the alleviation of the inflammatory processes of IBD [1].

In addition, metformin has an additional benefit of inhibiting the mammalian target of rapamycin (mTOR) [74], which is activated in patients with IBD and may be responsible for triggering the inflammatory process in IBD [82]. An early study showed that rosiglitazone might activate the mTOR signaling pathway, which mediates its adipogenic effect [83]. Another recent study confirmed the involvement of mTOR in rosiglitazone’s regulation of adiponectin production and secretion and the oxidative metabolism of branched-chain amino acids [84]. Therefore, the anti-inflammatory benefits following the use of TZDs might have further been mitigated by their activation of the mTOR pathway which is actually inhibited by the use of metformin.

Our recent study suggested that metformin plays an important role in the inhibition of immune-mediated skin diseases including urticaria, allergic contact dermatitis, and psoriasis [85]. This observation provides a clue that metformin might also be able to modulate the autoimmune processes of IBD.

Because metformin [74] and TZDs [86] share similar effects on the improvement of insulin resistance and reduction of blood glucose, these metabolic actions may not satisfactorily explain the discrepant findings between these two classes of drugs.

Taken together, it seems reasonable to suggest that rectifying the PPARγ signaling pathways by TZDs, even if they really work, would not actually prevent the development of IBD clinically. On the other hand, the use of metformin may affect the development of IBD, probably through its exceptional ability to change the gut microbiota, to maintain or reduce body weight, to modulate autoimmunity, and to inhibit the mTOR pathway. All of these pleiotropic effects of metformin may collectively contribute to a significant and sustained alleviation of the inflammatory processes of IBD. The improvement in insulin resistance and glycemic control associated with the use of either metformin or TZDs may not be responsible for the discrepant findings observed in the development of IBD between these two classes of antidiabetic drugs.

### 3.4. Implications

There are several clinical implications from the present study. First, the potential benefits of rosiglitazone on IBD derived from preclinical studies should not be immediately interpreted as a potential usefulness in the treatment of human IBD; at least, our present study did not favor such a benefit of rosiglitazone. Together with the finding of a null association with pioglitazone [57], the usefulness of TZDs in the prevention or treatment of IBD in humans is not very optimistic, at least in their current formulations.

Second, because we found a potential benefit of rosiglitazone on UC but not on CD in the secondary analyses when IBD was analyzed for UC and CD separately, we were interested to know whether similar findings could be seen in patients treated with pioglitazone. We additionally analyzed the data in association with pioglitazone use (this was not conducted in our previously published paper [57]) and found that pioglitazone had a null effect on either UC or CD (data not shown). Therefore, future research should focus on more detailed analyses on the potential benefit of rosiglitazone on UC by enrolling enough case numbers of UC and with more adequate consideration of potential confounders.

Third, although rosiglitazone does not cause hypoglycemia and may have some potential benefits in the risk reduction of some cancers [65], it potentially increases the risk of heart failure [87] and cardiovascular disease [55]. Therefore, the reuse of rosiglitazone as an antidiabetic drug for treating hyperglycemia in patients with type 2 diabetes mellitus needs additional research to balance the potential risk and benefit.

### 3.5. Strengths

The use of a nationwide database that covers >99% of Taiwan’s population may have some inherent merits. First, because of a lower risk of selection bias, we believe that the generalization of the findings to the whole population might be more confident. Second, the risk of information bias resulting from self-reporting was minimal because of the use of the objective medical records documented in the database. Third, it is less likely to have detection bias as a result of different socioeconomic status in the study because the healthcare services provided in the NHI system require very low drug cost-sharing and the copayment can even be waived when the patients receive prescription refills for chronic disease or when the patients have a low-income household or when the patients are veterans. Finally, we considered the timeframes of enrollment of patients and follow-up period by taking into account the potential psychological impacts of the attending physicians leading to their behavioral changes in the prescription of the drug and the possible nonadherence to the drug by the patients even when it had been prescribed when the issue of a potential risk of cardiovascular disease might be induced by rosiglitazone was brought up [55].

### 3.6. Limitations

The following study limitations must be considered. First, most confounders in the database were not collected primarily and we did not have measurement data such as family history, genetic parameters, lifestyle, smoking, alcohol drinking, anthropometric factors, dietary pattern, and nutritional status.

Second, biochemical data of levels of inflammatory cytokines, C-reactive protein, fecal calprotectin, glucose, and insulin were not available for analyses in the database.

Third, it should be mentioned that unmeasured confounders can never be statistically adjusted for. Therefore, we could not exclude the possible existence of some important confounders that might have influenced the results.

Fourth, because the average follow-up time was approximately 4.4 years in either the never users or the ever users (Table 2), we did not know whether such a relatively short period of time would be sufficient to capture the long-term effects of rosiglitazone on the risk of IBD.

Fifth, we did not have clinical information and laboratory data to investigate the severity of IBD in the patients.

Sixth, because of the lack of colonoscopic examination for the diagnosis of the outcome, misdiagnosis in some patients was possible. However, if the misclassification was not differential, we would expect a bias toward the null in the estimated hazard ratios [88,89].

Seventh, as mentioned earlier, we could not exclude the possibility of a lack of statistical power and the potential benefit of rosiglitazone on UC in secondary analysis. We think that future investigation with a request of a larger database of the NHI is warranted.

Finally, because this is a retrospective matched cohort study, the interpretation of the results in terms of causal inference should be cautious and future prospective cohort studies or clinical trials are needed to confirm our findings.

Because of these potential limitations, the conclusions of the present work may not be directly extrapolated to clinical situations.

## 4. Materials and Methods

### 4.1. Taiwan’s National Health Insurance

Taiwan has implemented a nationwide and compulsory healthcare insurance since 1 March 1995. This healthcare insurance is called the NHI and it covers more than 99.6% of Taiwan’s population. To provide comprehensive and equal medical care to the insurants, the Bureau of the NHI has contracted with all hospitals and more than 93% of all medical settings in Taiwan. All medical records and reimbursement information, including disease diagnoses, medication prescriptions, and performed procedures, are stored in computerized files before submitting for reimbursement. Researchers can apply for academic use of the database after ethics review and approval. This study was approved by the Research Ethics Committee of the National Health Research Institutes with an approval number NHIRD-102-175.

Throughout the research period, the disease coding system used in the database was the International Classification of Diseases, Ninth Revision, Clinical Modification (ICD-9-CM). We used the codes of 250.XX as a diagnosis of diabetes mellitus. The codes of 555 (regional enteritis or CD) and/or 556 (ulcerative enterocolitis or UC) were used for IBD diagnosis, as previously used in our study investigating the effect of pioglitazone [57].

### 4.2. Enrollment of Study Subjects

**Figure 1:**
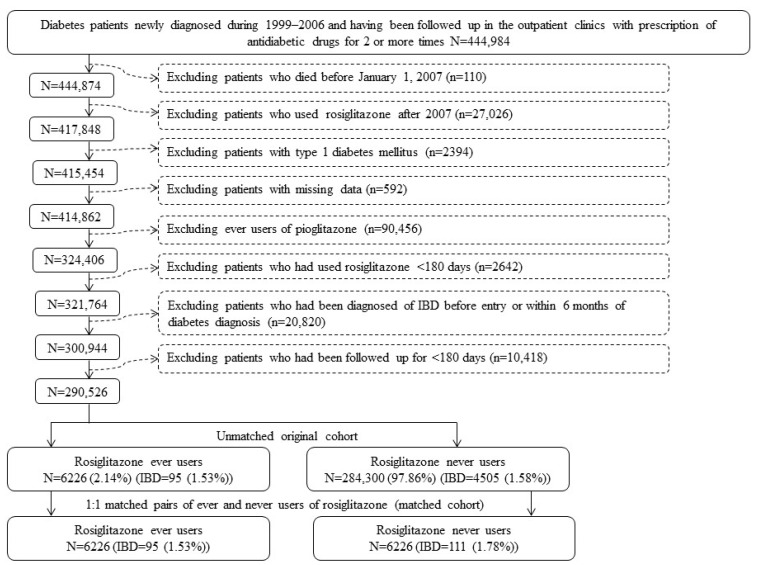
The procedures followed in the enrollment of study subjects. IBD: inflammatory bowel disease.

This was a retrospective cohort study. We enrolled from the NHI database a cohort of 1:1 matched pairs of ever users and never users of rosiglitazone based on PS. The flowchart in Figure 1 shows the procedures that we followed in the enrollment of the subjects used for analyses. At first, we identified 444,984 new-onset diabetes patients within the period from 1999 to 2006. We did not include patients who had a diagnosis of diabetes mellitus between 1996 and 1998 to ensure that the patients’ diagnosis of diabetes mellitus was made after 1999. To confirm a correct diagnosis of diabetes mellitus, the enrolled patients should have received prescriptions of antidiabetic drugs for at least two times at the outpatient clinics. We tried to maximize from the available database as many eligible patients as possible for follow-up and therefore we restricted the exclusion criteria to a minimum without unnecessary exclusions of eligible patients according to the steps shown in Figure 1. As a result, we identified an unmatched cohort consisting of 6226 ever users and 284,300 never users of rosiglitazone. Logistic regression that included all characteristics listed in Table 1 as independent variables was then used to create PS. The Greedy 8 → 1 digit match algorithm proposed by Parson [90] was then used to create a matched cohort consisting of 6226 ever users and 6226 never users.

In Taiwan, we have had only two drugs, namely, rosiglitazone and pioglitazone, marketed in the class of TZD. Following the challenge of a potential risk of cardiovascular disease associated with the use of rosiglitazone in a meta-analysis published in 2007 [55], rosiglitazone has been withdrawn from the markets of many countries, including Taiwan. To avoid the potential impact of some unknown factors following the publication of this meta-analysis, we restricted our analyses by enrolling patients of ever users of rosiglitazone based on the prescription of the drug before 2007 and excluding those who had a prescription of rosiglitazone after 2007 (Figure 1).

Users of pioglitazone were deliberately excluded for analyses (Figure 1) because of the following reasons:

Besides the glucose lowering effect, very different safety profiles in terms of cardiovascular disease, cancer, and dementia have been shown between rosiglitazone and pioglitazone. For example, rosiglitazone may have a potential risk of myocardial infarction and cardiovascular death [55]. On the other hand, pioglitazone may significantly improve lipid profiles [62], reduce cardiovascular events in patients with type 2 diabetes mellitus [91], and prevent secondary stroke in patients with insulin resistance and a previous ischemic stroke [92]. With regard to cancer, a potential risk of bladder cancer has been reported in patients who had been treated with pioglitazone, as shown in the interim analysis of the Kaiser Permanente Northern California study published in 2011 [56]. Furthermore, rosiglitazone significantly reduces the risk of thyroid cancer [65], but pioglitazone shows a null association with thyroid cancer [67]. Pioglitazone and rosiglitazone also show different effects on their association with dementia. As shown in our previous observational studies, a significantly lower risk of dementia was associated with pioglitazone [66] but not with rosiglitazone [64]. Therefore, rosiglitazone and pioglitazone should be viewed as two different entities when we intend to analyze the safety profile or the risk associated with cancer or noncancer diseases.

### 4.3. Potential Confounders and Statistical Analyses

Potential confounders are shown in Table 1. The ICD-9-CM codes for the related diagnoses have been reported previously [57].

The matched cohort was used for statistical analyses with the aid of the SAS statistical software, version 9.4 (SAS Institute, Cary, NC, USA). A *p* < 0.05 was used as a cutoff for indicating statistical significance.

Standardized difference was calculated for each covariate listed in Table 1 to examine the potential risk of confounding by indication. We used a cutoff value of >10% to indicate the potential existence of such a confounding from the variable. This is the generally recommended cutoff value by most investigators [93].

We calculated the cumulative duration in months and cumulative dose in mg of rosiglitazone therapy and categorized ever users according to the tertiles of these parameters for the assessment of a dose–response relationship. We calculated incidence density of IBD with regard to rosiglitazone exposure in never users, ever users, and subgroups of ever users categorized by the tertiles of cumulative duration and cumulative dose. We identified newly diagnosed cases of IBD during follow-up with regard to the different subgroups of rosiglitazone exposure. The numerators of the incidence density were the case numbers of newly diagnosed IBD in the respective subgroups. The denominators of the incidence density were the person-years of follow-up in the respective subgroups. We set the date of start of follow-up as 1 January 2007 and the patients were then followed up until 31 December 2011 when a new diagnosis of IBD was made, or the last reimbursement record, or the date of death, whichever occurred first.

For primary analyses, we estimated hazard ratios and their 95% confidence intervals for IBD by Cox proportional hazards regression model incorporated with the inverse probability of treatment weighting using the PS. In comparison to the never users, hazard ratios were estimated for ever users and for each tertile of cumulative duration and cumulative dose. In consideration that CD and UC may have different characteristics and clinical patterns, we also estimated the hazard ratios for CD and UC separately as secondary analyses.

We also evaluated the joint effects of rosiglitazone and some major risk factors of IBD by using the traditional Cox regression after adjustment for all covariates listed in Table 1. These major risk factors included psoriasis/arthropathies, dorsopathies (ankylosing spondylitis is associated with IBD [94]), and chronic obstructive pulmonary disease/tobacco abuse (as a surrogate marker for smoking that can affect IBD [2,95]). The joint effects were evaluated by estimating hazard ratios with regard to the presence and absence of risk factors and rosiglitazone use in the following subgroups: (1) risk factor (+)/rosiglitazone (-) as the referent group; (2) risk factor (+)/rosiglitazone (+); (3) risk factor (-)/rosiglitazone (-); and (4) risk factor (-)/rosiglitazone (+). We also estimated the value of *P*-interaction for each model.

Because we previously showed that metformin may reduce the risk of IBD [68], we additionally investigated the joint effect of and interaction between metformin and rosiglitazone.

## 5. Conclusions

The findings of the present study suggest that rosiglitazone does not affect the risk of IBD and that rosiglitazone does not interact with major risk factors or metformin in the development of IBD in patients with type 2 diabetes mellitus. However, we cannot exclude the possible benefit of rosiglitazone on UC in secondary analyses. Because this is an observational study that may have potential limitations including a lack of sufficient power (especially the small case numbers of UC) and an inability to consider all confounders, we acknowledge that further confirmation of the null effect of rosiglitazone on IBD observed in the present study is recommended. Personalized medicine [27] and application of nanotechnology [59,96,97] and artificial intelligence [98] may help to identify patients at a high risk of developing IBD and its related complications, and to identify subgroups of patients who can benefit from rosiglitazone treatment. These novel technologies should be incorporated in future research.

## Figures and Tables

**Table 1:** Characteristics of enrolled subjects with regard to rosiglitazone exposure in a propensity-score-matched cohort.

Variable	Never Users of Rosiglitazone		Ever Users of Rosiglitazone		*p* Value	Standardized Difference
	(*n* = 6226)		(*n* = 6226)			
	n	%	n	%		
Basic information						
Age * (years)	63.83	12.13	63.85	11.92	0.9488	−0.03
Sex (men)	3392	54.48	3330	53.49	0.2649	−2.10
Diabetes duration * (years)	5.69	2.18	5.69	2.07	0.9845	0.15
Occupation						
I	2638	42.37	2643	42.45	0.9402	
II	1276	20.49	1250	20.08		−1.01
III	1123	18.04	1127	18.10		0.32
IV	1189	19.10	1206	19.37		0.64
Living region						
Taipei	2531	40.65	2472	39.70	0.5279	
Northern	575	9.24	593	9.52		0.97
Central	1671	26.84	1734	27.85		2.42
Southern	644	10.34	659	10.58		0.87
Kao-Ping and Eastern	805	12.93	768	12.34		−1.85
Major comorbidities associated with diabetes mellitus						
Hypertension	5295	85.05	5290	84.97	0.9001	−0.25
Dyslipidemia	5168	83.01	5150	82.72	0.6686	−0.76
Obesity	319	5.12	340	5.46	0.4006	1.48
Diabetes-related complications						
Ischemic heart disease	3209	51.54	3153	50.64	0.3154	−1.87
Stroke	2185	35.09	2167	34.81	0.7351	−0.70
Peripheral arterial disease	1785	28.67	1785	28.67	0.6492	0.74
Diabetic polyneuropathy	2042	32.80	2093	33.62	0.3318	1.67
Eye disease	2480	39.83	2498	40.12	0.7419	0.61
Nephropathy	1942	31.19	1959	31.46	0.7426	0.48
Factors that might affect exposure/outcome						
Gingival and periodontal diseases	5393	86.62	5396	86.67	0.9370	0.22
Pulmonary tuberculosis	262	4.21	293	4.71	0.1782	2.26
Pneumonia	1106	17.76	1187	19.07	0.0611	3.27
Head injury	242	3.89	236	3.79	0.7796	−0.47
Dementia	502	8.06	521	8.37	0.5352	0.99
Parkinson’s disease	204	3.28	207	3.32	0.8804	0.32
Hypoglycemia	446	7.16	470	7.55	0.4100	1.40
Osteoporosis	1295	20.80	1338	21.49	0.3453	1.67
Human immunodeficiency virus infection	6	0.10	5	0.08	0.7629	−0.63
Cancer	1091	17.52	1093	17.56	0.9624	−0.01
Alcohol-related diagnoses	344	5.53	346	5.56	0.9376	0.22
Tobacco abuse	183	2.94	189	3.04	0.7521	0.47
Chronic obstructive pulmonary disease	3070	49.31	3111	49.97	0.4624	1.08
Heart failure	1379	22.15	1386	22.26	0.8800	0.18
Valvular heart disease	704	11.31	745	11.97	0.2519	2.03
Dorsopathies	4789	76.92	4790	76.94	0.9830	−0.02
Arthropathies and related disorders	4949	79.49	4913	78.91	0.4267	−1.50
Psoriasis and similar disorders	173	2.78	176	2.83	0.8706	0.26
Organ transplantation	44	0.71	43	0.69	0.9143	−0.15
Hepatitis B virus infection	250	4.02	234	3.76	0.4582	−1.27
Hepatitis C virus infection	294	4.72	268	4.30	0.2617	−1.89
Liver cirrhosis	247	3.97	212	3.41	0.0960	−2.86
Other chronic nonalcoholic liver diseases	553	8.88	583	9.36	0.3505	1.72
Antidiabetic drugs						
Insulin	259	4.16	260	4.18	0.9642	0.33
Sulfonylureas	4495	72.20	4432	71.19	0.2101	−2.43
Metformin	4111	66.03	4068	65.34	0.4170	−1.28
Meglitinide	419	6.73	432	6.94	0.6443	0.76
Acarbose	708	11.37	748	12.01	0.2646	2.15
Other commonly used medications						
Angiotensin converting enzyme inhibitors/Angiotensin receptor blockers	4932	79.22	4878	78.35	0.2366	−2.17
Calcium channel blockers	3863	62.05	3828	61.48	0.5187	−1.21
Statins	4672	75.04	4635	74.45	0.4454	−1.38
Fibrates	2676	42.98	2717	43.64	0.4584	1.29
Aspirin	4183	67.19	4201	67.48	0.7309	0.59
Corticosteroids	251	4.03	241	3.87	0.6455	−0.67

* Age and diabetes duration are expressed as continuous variables in mean and standard deviation.

**Table 2:** Incident case numbers, incidence rates, and hazard ratios of inflammatory bowel disease in never users and in different subgroups of ever users of rosiglitazone.

Rosiglitazone Use	Number of Incident Case	Number of Cases Followed	Person-Years	Incidence Rate (per 100,000 Person-Years)	Hazard Ratio	95% Confidence Interval	*p* Value
Never users	111	6226	27,597.94	402.20	1.000		
Ever users	95	6226	27,235.66	348.81	0.870	(0.661–1.144)	0.3174
Tertiles of cumulative duration of rosiglitazone therapy (months)							
Never users	111	6226	27,597.94	402.20	1.000		
<12.4	29	2056	8782.12	330.22	0.826	(0.549–1.244)	0.3605
12.4–25.3	28	2054	8999.12	311.14	0.776	(0.513–1.174)	0.2301
>25.3	38	2116	9454.43	401.93	0.996	(0.689–1.440)	0.9843
Tertiles of cumulative dose of rosiglitazone therapy (mg)							
Never users	111	6226	27,597.94	402.20	1.000		
<1624	30	2044	8875.26	338.02	0.844	(0.564–1.263)	0.4090
1624–3596	32	2065	9179.81	348.59	0.866	(0.584–1.283)	0.4723
>3596	33	2117	9180.59	359.45	0.897	(0.608–1.323)	0.5844

**Table 3:** Joint effects of and interactions between rosiglitazone and risk factors/metformin use.

Risk Factor/Rosiglitazone Use	Incident Case Number	Cases Followed	Person-Years	Incidence Rate (per 100,000 Person-Years)	Hazard Ratio	95% Confidence Interval	*p* Value
Psoriasis/Arthropathies (+)/Rosiglitazone (-)	98	4979	22,081.13	443.82	1.000		
Psoriasis/Arthropathies (+)/Rosiglitazone (+)	81	4948	21,709.31	373.11	0.828	(0.616–1.112)	0.2097
Psoriasis/Arthropathies (-)/Rosiglitazone (-)	13	1247	5516.81	235.64	0.523	(0.287–0.954)	0.0345
Psoriasis/Arthropathies (-)/Rosiglitazone (+)	14	1278	5526.35	253.33	0.571	(0.319–1.023)	0.0595
*P*-interaction							0.3103
Dorsopathies (+)/Rosiglitazone (-)	92	4789	21,291.34	432.10	1.000		
Dorsopathies (+)/Rosiglitazone (+)	73	4790	21,003.45	347.56	0.796	(0.584–1.083)	0.1463
Dorsopathies (-)/Rosiglitazone (-)	19	1437	6306.60	301.27	0.846	(0.508–1.410)	0.5214
Dorsopathies (-)/Rosiglitazone (+)	22	1436	6232.21	353.00	0.986	(0.608–1.597)	0.9533
*P*-interaction							0.2777
COPD/Tobacco abuse (+)/Rosiglitazone (-)	62	3143	13,853.61	447.54	1.000		
COPD/Tobacco abuse (+)/Rosiglitazone (+)	46	3181	13,834.35	332.51	0.733	(0.500–1.076)	0.1125
COPD/Tobacco abuse (-)/Rosiglitazone (-)	49	3083	13,744.33	356.51	0.788	(0.534–1.163)	0.2304
COPD/Tobacco abuse (-)/Rosiglitazone (+)	49	3045	13,401.32	365.64	0.802	(0.544–1.182)	0.2647
*P*-interaction							0.7492
Metformin (-)/Rosiglitazone (-)	40	2115	9177.49	435.85	1.000		
Metformin (-)/Rosiglitazone (+)	34	2158	9383.34	362.34	0.789	(0.495–1.258)	0.3201
Metformin (+)/Rosiglitazone (-)	71	4111	18,420.45	385.44	0.798	(0.535–1.191)	0.2694
Metformin (+)/Rosiglitazone (+)	61	4068	17,852.32	341.69	0.718	(0.477–1.081)	0.1128
*P*-interaction							0.6761

COPD: chronic obstructive pulmonary disease.

## Data Availability

The datasets in this article are not readily available because local regulations restrict public availability of the dataset to protect privacy. Requests to access the datasets should be directed to C.T., ccktsh@ms6.hinet.net.
